# *Wolbachia* Strain *w*Gri From the Tea Geometrid Moth *Ectropis grisescens* Contributes to Its Host’s Fecundity

**DOI:** 10.3389/fmicb.2021.694466

**Published:** 2021-07-19

**Authors:** Yong Zhang, Song Liu, Rui Jiang, Chen Zhang, Tian Gao, Yun Wang, Cui Liu, Yanhua Long, Yinglao Zhang, Yunqiu Yang

**Affiliations:** ^1^State Key Laboratory of Tea Plant Biology and Utilization, Anhui Agricultural University, Hefei, China; ^2^School of Life Sciences, Anhui Agricultural University, Hefei, China; ^3^Lu’an Academy of Agricultural Sciences, Lu’an, China

**Keywords:** cytoplasmic incompatibility, culture-independent, mutualism, reciprocal cross, multilocus sequence types

## Abstract

Members of the *Wolbachia* genus manipulate insect–host reproduction and are the most abundant bacterial endosymbionts of insects. The tea Geometrid moth *Ectropis grisescens* (Warren) (Lepidoptera: Geometridae) is the most devastating insect pest of tea plants [*Camellia sinensis* (L.) O. Kuntze] in China. However, limited data on the diversity, typing, or phenotypes of *Wolbachia* in *E. grisescens* are available. Here, we used a culture-independent method to compare the gut bacteria of *E. grisescens* and other tea Geometridae moths. The results showed that the composition of core gut bacteria in larvae of the three Geometridae moth species was similar, except for the presence of *Wolbachia*. Moreover, *Wolbachia* was also present in adult female *E. grisescens* samples. A *Wolbachia* strain was isolated from *E. grisescens* and designated as *w*Gri. Comparative analyses showed that this strain shared multilocus sequence types and *Wolbachia* surface protein hypervariable region profiles with cytoplasmic incompatibility (CI)-inducing strains in supergroup B; however, the *w*Gri-associated phenotypes were undetermined. A reciprocal cross analysis showed that *Wolbachia*-uninfected females mated with infected males resulted in 100% embryo mortality (0% eggs hatched per female). Eggs produced by mating between uninfected males and infected females hatched normally. These findings indicated that *w*Gri induces strong unidirectional CI in *E. grisescens*. Additionally, compared with uninfected females, *Wolbachia*-infected females produced approximately 30–40% more eggs. Together, these results show that this *Wolbachia* strain induces reproductive CI in *E. grisescens* and enhances the fecundity of its female host. We also demonstrated that *w*Gri potential influences reproductive communication between *E. grisescens* and *Ectropis obliqua* through CI.

## Introduction

*Wolbachia* are Gram-negative bacteria that were first identified in the oophoron of *Culex pipiens*. Their prevalence in arthropods and nematodes worldwide and stunning arsenal of parasitic and mutualistic adaptations (Zug and Hammerstein, 2012). It has been recently estimated that ∼50% of arthropod and several filarial nematode species harbor *Wolbachia* (Weinert et al., 2015; Lefoulon et al., 2016). Moreover, *Wolbachia* is estimated to infect 40–60% of insect species (Mateos et al., 2020). In fact, *Wolbachia* is the most common and widespread facultative symbiont of insects (Zug and Hammerstein, 2015). As parasites, *Wolbachia* manipulate insect–host reproduction in various ways, *via* cytoplasmic incompatibility (CI), male-killing, feminization, and parthenogenesis, in order to facilitate their maternal transmission (Yen and Barr, 1971; Rousset et al., 1992). Of these, CI is the most prevalent host phenotype caused by the presence of *Wolbachia* (Stouthamer et al., 1999). In contrast to *Wolbachia*’s well-established reproductive role, recent years have extensive evidence that *Wolbachia* can benefit their arthropod host as mutualists (Teixeira et al., 2008; Brownlie et al., 2009; Nikoh et al., 2014; Zug and Hammerstein, 2015). In some insects, *Wolbachia* strains function as mutualists to provide benefits to the host, including supplementing essential nutrients, protection of the host against pathogens, increasing successful egg development, and enhancing female insect host fecundity (Dedeine et al., 2001; Martijn and Ellers, 2008; Hosokawa et al., 2010; Iturbe-Ormaetxe et al., 2011; Guo et al., 2018). For example, *Wolbachia* provides riboflavin (vitamin B_2_) to all its insect host, and *Wolbachia*’s riboflavin provisioning certainly contributes to growth, survival, and reproduction in its insect host (Moriyama et al., 2015). For instance, in the two planthopper species *Nilaparvata lugens* and *Laodelphax striatellus*, intracellular *Wolbachia* strains *w*Lug and *w*StriCN (both in supergroup B) provide biotin and riboflavin to enhance reproduction of their female hosts (Ju et al., 2020). Accurate identification of *Wolbachia* strains is a fundamental component of all *Wolbachia*-related research. However, *Wolbachia* cannot be cultured outside host cells, and identification of *Wolbachia* strain is solely based on molecular typing.

The genus *Wolbachia* is currently divided into at least 17 possible phylogenetic supergroups, named A–F, H–Q, and S (O’Neill et al., 1992; Werren et al., 1995; Bandi et al., 1998; Ros et al., 2009; Lefoulon et al., 2020). Supergroups A and B are primarily found in arthropods and often function as reproductive parasites that manipulate host reproduction to increase their own spread through the matriline (Kaur et al., 2021). An earlier phylogenetic analysis based on *Wolbachia* surface protein (*wsp*) gene sequences further divided the A and B supergroups into 12 groups (Mel, AlbA, Pip, Uni, Riv, Pap, Aus, Ori, Dei, Haw, Con, and Mors) (Zhou et al., 1998). Later, seven more groups were added (Kue, Dro, Sib, Kay, Div, For, and Vul) (van Meer et al., 1999). However, the *wsp* gene in different strains of *Wolbachia* has undergone extensive recombination (Werren and Bartos, 2001). Thus, this single-locus identification system is unreliable as a research tool. A multilocus sequence typing (MLST) system was developed to type *Wolbachia* strains of diverse origins (Baldo et al., 2006). This system is based on five conserved genes (*gatB*, *coxA*, *hcpA*, *ftsZ*, and *fbpA*) and four WSP hypervariable regions (HVRs): HVR1 (52–84 aa), HVR2 (85–134 aa), HVR3 (135–185 aa), and HVR4 (186–222 aa). Data generated using the five gene-based MLST system have been deposited in a web-accessible database^1^ that facilitates the storage, management, and analysis of sequence data and isolate information.

Tea plant [*Camellia sinensis* (L.) O. Kuntze], a perennial evergreen woody plant, is one of the most important economic crops in almost 30 countries such as China, Japan, India, Kenya, and Indonesia. The young leaves and buds are processed into a variety of tea, including green, white, yellow, oolong, black, and dark tea, which is a popularly daily beverage (Jiang et al., 2019). Tea has numerous health and medical benefits for humans due to its many characteristic secondary plant metabolites, including catechins, caffeine, polyphenols, and theanine (Too et al., 2015; Ji et al., 2016). Tea production plays a role in the development of agricultural economy in China. Unfortunately, leaf herbivory on tea plants by tea Geometridae moths can cause severe yield loss and quality damage for tea.

Tea Geometridae moths are the most destructive chewing insects for tea plant by feeding on the young tea leaves in China (Zhang, 2001). Among them, *Ectropis grisescens* (Warren) (Lepidoptera: Geometridae, Ennominae) is the most serious Geometridae pest of tea in China because of its wide distribution and destructive nature (Zhang et al., 2019; Pan et al., 2021). This pest infests thousands of hectares of tea plants per year, severely reducing tea plant growth and negatively impacting tea production (Jiang et al., 2014). Compared with *Ectropis obliqua* and *Scopula subpunctaria*, the other main Geometridae pests of tea, *E. grisescens*, has a wider distribution and stronger fecundity (Chen L. L. et al., 2020). Little is known about why *E. grisescens* is the most harmful pest in tea gardens, and there is no explanation from the perspective of insect symbionts. Recently, extensive evidence shows that insect symbionts do affect essential physiological functions in their lepidoptera host, including degrading complex dietary polymers, supplementing essential nutrients, overcoming plant anti-herbivore defenses, or strengthening of immune responses for protection against pathogens (Paniagua et al., 2018). A study reported that mundticin KS (bacteriocin) produced by gut bacteria *Enterococcus mundtii* inhibits pathogen colonization in its host *Spodoptera littoralis* (Lepidoptera: Noctuidae) (Shao et al., 2017). Besides, Lei et al. (2020) found that *Wolbachia* reduce the susceptibility of striped stem borer, *Chilo suppressalis* (Lepidoptera: Crambidae), to two insecticides (fipronil and avermectin).

In a previous study, we detected *Wolbachia* in the gut of *E. grisescens* larvae (Zhang et al., 2019). However, the *Wolbachia* strain in *E. grisescens* was not typed, and its resulting phenotypes were not elucidated. Here, we use a culture-independent method to investigate and compare the diversity and richness of gut bacterial communities among three species of Geometridae moths and found that the presence of *Wolbachia* was the most obvious difference. MLST- and wsp-based phylogenetic analyses revealed that the *Wolbachia* from different populations of *E. grisescens* had the same *Wolbachia*, which is from supergroup B. Reciprocal cross analyses revealed that *Wolbachia* enhances the fecundity of *E. grisescens*. This may be one of the reasons why its host *E. grisescens* is the most damaging leaf-eating tea pest in China.

## Materials and Methods

### Insect Collection and Preservation

The larvae were collected from tea gardens in the five main tea-producing provinces of China (Table 1) and were maintained at the State Key Laboratory of Tea Plant Biology and Utilization, Anhui Agricultural University, Hefei, China (31.86°N, 117.27°E). The collected larvae were reared on tea leaves in transparent boxes in a controlled climate room (22°C ± 1°C; relative humidity 75% ± 10%; 16-h light:8-h dark photoperiod). The tea leaves used in the experiments were cut from branches of tea plants using scissors and inserted into floral foam for storage.

**TABLE 1:** Collection information for larvae samples.

Insect	Sampling locality	Latitude/longitude	Sampling time
*Ectropis grisescens*	Luan, Anhui, China	31.47°N, 116.93°E	September 2016
*Ectropis grisescens*	Xishui, Hubei, China	30.28°N, 113.23°E	September 2020
*Ectropis grisescens*	Xinchang, Zhejiang, China	29.49°N, 120.99°E	June 2020
*Ectropis grisescens*	Nanping, Fujian, China	27.65°N, 117.98°E	September 2020
*Ectropis grisescens*	Menghai, Yunnan, China	24.98°N, 102.72°E	May 2020
*Ectropis obliqua*	Xuancheng, Anhui, China	31.12°N, 119.18°E	September 2020
*Scopula subpunctaria*	Xuancheng, Anhui, China	31.12°N, 119.18°E	September 2020

### Sample Preparation and DNA Extraction

For Geometrid larvae, 10 fifth instar larvae were pooled to provide a biological replicate and five biological replicates were established per group. For *E. grisescens* adults, five female adults were pooled to provide a biological replicate and five biological replicates were established per group. Note that the DNA used for *Wolbachia*-typing was extracted from single female moth.

Larvae were surface-sterilized by dipping in 70% ethanol for 15 s and then rinsing twice with sterile water for 15 s each time. Dissecting scissors were used to cut laterally behind the head capsule, and the gut was removed from the cuticle with larval forceps. The whole gut including the gut contents was collected and placed in a 2.0-ml micro-centrifuge tube for DNA extraction.

Wings were removed from female moths, and other tissues were surface-sterilized by dipping in 70% ethanol for 15 s, followed by rinsing twice with sterile water for 15 s each time. The tissues were then ground for 2 min and placed in a 2.0-ml micro-centrifuge tube for DNA extraction.

Total genomic DNA was extracted from samples using QIAamp DNA Stool Mini Kit (Qiagen, Hilden, Germany). The quality of the extracted DNA was assessed by electrophoresis on a 1.2% (w/v) agarose gel. The genomic DNA extracted from bacteria in the larvae gut was subjected to amplification and sequencing.

The Genomic DNA extracted from bacteria in adult female moths was subjected to metagenomic and *Wolbachia*-typing analyses.

### Amplification and Sequencing of the V3–V4 Region of the 16S rRNA Gene

Gut bacteria from three Geometridae larvae were analyzed by sequencing the V3–V4 region of the 16S ribosomal RNA gene (16S rRNA), using the Illumina NovaSeq platform.

Genomic DNA samples were subjected to PCRs for amplification of the V3–V4 regions of the 16S rRNA with the universal primers 338F (5′-ACTCCTACGGGAGGCAGCAG-3′) and 806R (5′-GGACTACHVGGGTWTCTAAT-3′). All PCR reactions consisted of 15 μl of Phusion ^®^High-Fidelity PCR Master Mix (New England Biolabs, Beverly, MA, United States), 2 μM forward and reverse primers, and 10 ng of template DNA. The thermal cycling conditions were as follows: initial denaturation at 98°C for 1 min, followed by 30 cycles of denaturation at 98°C for 10 s, annealing at 50°C for 30 s, elongation at 72°C for 30 s, and final extension at 72°C for 5 min. Then, the mixture of PCR products was purified with a Gel Extraction Kit (Qiagen).

After PCR amplification, the samples were sequenced on the Illumina NovaSeq platform (Illumina, San Diego, CA, United States) and 250-bp paired-end reads were generated. All sequences are available as SRA files at the National Center for Biotechnology Information Sequence Read Archive database (NCBI-SRA) under bioProject PRJNA720281 (SRA accession numbers: SAMN18644841-843).

### Bioinformatics and Statistical Analysis

The sequences were analyzed using the QIIME software package and used to compare the relative abundance of bacterial taxa (Caporaso et al., 2010b). Operational taxonomic units (OUTs) were assigned at 97% similarity cutoff using UCLUST version 1.2.22 (Edgar, 2010). The representative sequence (the sequence with the highest relative abundance) of each OTU was selected to build the overall OTU table. The taxonomic classification of each microbial OTU was assigned by Ribosomal Database Project classifier PyNast using SILVA and UNITE as bacterial 16S rRNA databases (Caporaso et al., 2010a). Abundance values of OTUs were normalized using a standard value for the sample with the least sequences. Subsequent analyses of alpha diversity were performed using this normalized output.

The alpha- and beta-diversity indexes were calculated using QIIME V1.7.0. The alpha-diversity indexes were the Pielou evenness index (*J*) and the ACE richness index (Shannon and Weaver, 1949; Pielou, 1966; Chao et al., 1993). Rarefaction curves and Shannon index were used to verify the quality and depth of sampling. Principal coordinate analysis (PCoA) with weighted and unweighted UniFrac distance metrics was performed to detect differences in microbial community structures among the three Geometridae larvae (Lozupone and Knight, 2005). Analysis of similarity (ANOSIM) was carried out based on Bray–Curtis dissimilarity to assess the difference of microbial community structures (Clarke, 1993). Microbes that showed significant differences in relative abundance among different groups were identified using Metastats software^2^ with false discovery rate (FDR) (Paulson et al., 2011).

### Metagenomic Analysis of Endosymbionts in *E. grisescens* Female Moths

The DNA sample was fragmented by sonication to produce 350-bp fragments; then, these DNA fragments were end-polished, A-tailed, and ligated with the full-length adaptor for Illumina sequencing with further PCR amplification. Finally, PCR products were purified using the AMPure XP system. The libraries were analyzed to determine size distribution with an Agilent2100 Bioanalyzer and quantified using real-time PCR.

Clustering of the index-coded samples was performed using the cBot Cluster Generation System according to the manufacturer’s instructions. After cluster generation, the libraries were sequenced on the Illumina NovaSeq 6000 platform and paired-end reads were generated.

The raw data obtained from the Illumina NovaSeq sequencing platform using Readfq (V8)^3^ were processed to obtain clean data for subsequent analyses. The processing steps removed reads containing low-quality bases (default quality threshold value ≤ 38, default length 40 bp), reads containing a certain proportion of N (unspecified) bases (default length 10 bp), and reads containing adapter sequences (default length 15 bp). The raw data have been submitted to the NCBI-SRA under bioProject PRJNA720281 (SRA accession number: SAMN18644841).

Considering the possibility that samples may be polluted with host DNA, the clean data were used in BLAST searches against the host database using Bowtie2.2.4 software (Bowtie2.2.4)^4^ to filter reads of host origin. The parameters were as follows : –end-to-end, –sensitive, -I 200, -X 400.

Due to serious host pollution, we use a read-based (mapping) approach for metagenomic analysis. Species annotations were based on clean data and the RPKM method was used to calculate abundance. The MetaPhalAn2 method was used to analyze bacterial diversity (Segata et al., 2012).

### Cloning and Sequencing of *wsp* and MLST Genes

The WSP locus was amplified using the *wsp* primers (*wsp*_F1: 5′-GTCCAATARSTGATGARGAAAC-3′ and *wsp*_R1: 5′-CYGCACCAAYAGYRCTRTAAA-3′) to confirm single infections. The PCR reactions were performed in 25-μl final volumes containing 9.7 μl of ddH_2_O, 12.5 μl of Premix Taq (LA Taq Version 2.0, TaKaRa, Dalian, China), 0.8 μl of DNA template, and 1 μl of each primer (10 μM). The thermal cycling conditions were as follows: 95°C for 3 min; 35 cycles of 94°C for 1 min, 54°C for 1 min, and 72°C for 90 s; 72°C for 10 min. The products were stored at 4°C.

The MLST loci were amplified in accordance with previously published protocols (see text footnote 1). The PCR reactions were performed in 25-μl final volumes containing 9.7 μl of ddH_2_O, 12.5 μl of Premix Taq (LA Taq Version 2.0, TaKaRa), 0.8 μl of DNA template, and 1 μl of each primer (10 μM). The thermal cycling conditions were as follows: 94°C for 4 min; 35 cycles of 94°C for 30 s, appropriate *T*_*m*_ (Table 2) for 45 s and 72°C for 90 s; 72°C for 10 min. The products were stored at 4°C.

**TABLE 2:** Primers used for PCR amplification and sequencing.

Gene	Primer	Sequence (5′-3′)	Annealing *T* (°C)
*gatB*	gatB_F1	GAKTTAAAYCGYGCAGGBGTT	54
	gatB_R1	TGGYAAYTCRGGYAAAGATGA	
*coxA*	coxA_F1	TTGGRGCRATYAACTTTATAG	55
	coxA_R1	CTAAAGACTTTKACRCCAGT	
*hcpA*	hcpA_F1	GAAATARCAGTTGCTGCAAA	55
	hcpA_R1	GAAAGTYRAGCAAGYTCTG	
*ftsZ*	ftsZ_F1	ATYATGGARCATATAAARGATAG	50
	ftsZ_R1	TCRAGYAATGGATTRGATAT	
*fbpA*	fbpA_F1	GCTGCTCCRCTTGGYWTGAT	53
	fbpA_R1	CCRCCAGARAAAAYYACTATTC	

Each sample was mixed with the same volume of 1× loading buffer containing SYBR green and then separated by electrophoresis on 1.2% (v/v) agarose gels for amplicon detection. All the PCR products were sequenced by Sangon Biotech (Shanghai, China). The sequences of all products were used in BLAST or MLST analyses at the GenBank and PubMLST databases, respectively.

### *Wolbachia* Strain Typing Based on *wsp* Gene

The amplified *wsp* gene sequences were subjected to BLAST analysis at the GenBank database. A list of *Wolbachia* isolates, including those reported by Zhou et al. (1998) and van Meer et al. (1999) and those with *wsp* gene sequences in GenBank, was created (Supplementary Table 1). These isolates were used as references to classify the type of *Wolbachia* strain isolated from *E. grisescens*.

A phylogenetic tree was constructed using the *Wolbachia wsp* genes and the maximum likelihood (ML) approach implemented in MEGA X. The substitution model was Tamura 3-parameter and gamma distributed (G) with 1000 bootstrap replications.

### MLST, WSP HVR Profiling, and Phylogenetic Analyses

The sequences of *gatB*, *coxA*, *hcpA*, *ftsZ*, *fbpA*, and *wsp* were submitted to the PubMLST database for sequence typing to generate MLST allelic profiles and WSP HVR profiles. A list of *Wolbachia* isolates was generated by searching PubMLST and downloading isolate sequences from GenBank (Supplementary Table 2). Isolates with a complete set of MLST and HVR profiles, as well as phenotypic data, were selected. These isolates had been used by Baldo et al. (2006) to establish the MLST typing system for *Wolbachia*. The ML trees were constructed using the concatenated MLST and *wsp* sequences, independently, in MEGA X.

The alignments of concatenated MLST alleles and *wsp* alleles were generated using ClustalW with 1000 bootstrap iterations. For phylogenetic reconstructions, the nucleotide substitution model was determined by the BIC value calculated by MEGAX software. The best-fit substitution models were as follows: GTR + G for MLST and T92 + G for *wsp*. The sequences of the *wsp* gene and the five genes used for MLST of the *Wolbachia* strain from *E. grisescens* have been deposited in the GenBank database (Accession number: MW630082-MW630111).

### Influence of *Wolbachia* on *E. grisescens* Phenotypes

To analyze the effects of *Wolbachia* on its host *E. grisescens*, the fecundity (egg production) of infected (*W*^+^) and uninfected (*W*^–^) insects was recorded. All the insects used in the experiments were confirmed to be infected with *Wolbachia* by amplifying the *wsp* gene. *E. grisescens* was collected from Lu’an, Anhui Province, and successfully subcultured for more than 40 generations.

The F1 generation of *W*^+^ *E. grisescens*, as larvae, were reared on tea leaves until they reached the third instar stage. Then, 140 healthy third-instar larvae were divided into two groups of 70 larvae, which were placed individually in 90-mm plastic Petri dishes. Each dish contained food for the insects and had a tight lid. The control group was fed an agar-based insect diet, and the antibiotic group was fed the insect diet supplemented with 300 μg/ml each of rifampicin, gentamicin, penicillin, and streptomycin (Zhang et al., 2019). The insect diet comprised 60 ml of sterile water, 20 g of tea powder, and 2 g of wheat agar. Fresh tea leaves were collected, freeze-dried, and ground to make the tea powder.

To ensure that the effects on reproduction were due to the absence of *Wolbachia* and not due to antibiotics, the same antibiotics were used to treat *E. obliqua* and *S. subpunctaria*.

### Reciprocal Cross Analysis

*Ectropis obliqua* and *E. grisescens* are two closely related moth species. Reciprocal cross analyses were conducted to determine whether *Wolbachia* affects reproduction between these two moths. *E. grisescens* was collected from Luan, Anhui Province, and successfully subcultured for more than 40 generations. *E. obliqua* was collected from Xuancheng, Anhui Province, and successfully subcultured for more than three generations.

We selected newly emerged moths of *E. obliqua* and *E. grisescens* for reciprocal crosses. The egg hatching rate was counted.

### Data Analyses

GraphPad Prism 7.0, R, or Origin 9.0 was used for statistical analyses and to construct figures.

## Results

### Composition and Diversity of Gut Bacteria in Larvae of Three Tea Geometridae

**FIGURE 1:**
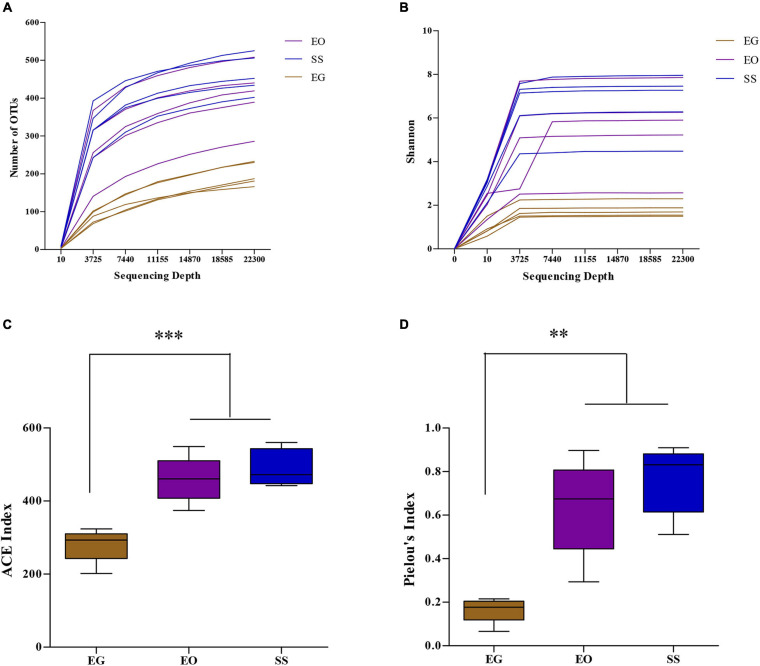
Rarefaction curves, based on number of operational taxonomic units (OTUs) observed **(A)**, and Shannon index **(B)**, and the alpha-diversity, based on ACE index **(C)**, and Pielou’s index **(D)**, of the microbial community in the larval gut of *E. grisescens*, *E. obliqua*, and *S. subpunctaria* (EG, EO, and SS, respectively). Significant differences were detected by unpaired two-tailed *t*-test; **p < 0.001; ***p < 0.0001.

The Illumina NovaSeq sequencing of the bacterial 16S rRNA amplicons from larvae of *E. grisescens* (EG), *E. obliqua* (EO), and *S. subpunctaria* (SS) yielded 951,717 raw reads in total. After quality filtering and read merging, a total of 876,703 high-quality sequences remained for bacteria. Rarefaction curves (Figure 1A) and Shannon index (Figure 1B) clearly demonstrated that the sampling efforts were adequate to fully represent the richness of the gut communities analyzed.

The OTU richness (ACE index) of the gut bacteria of EG (280.04 ± 41.54) differed from those of EO (458.75 ± 55.42; *t* = 6.91; df = 7; *p* < 0.0001) and SS (490.30 ± 45.15; *t* = 6.91; df = 7; *p* < 0.0001) (Figure 1C). The OTU evenness (Pielou index; *J*) of the gut bacteria of EG (*J* = 0.16 ± 0.05) differed from those of EO (*J* = 0.64 ± 0.19; *t* = 4.62; df = 8; *p* < 0.001) and SS (*J* = 0.76 ± 0.14; *t* = 7.98; df = 8; *p* < 0.0001) (Figure 1D). Thus, the OTU richness and evenness of the gut bacteria were lowest in *E. grisescens* among the three species analyzed.

**FIGURE 2:**
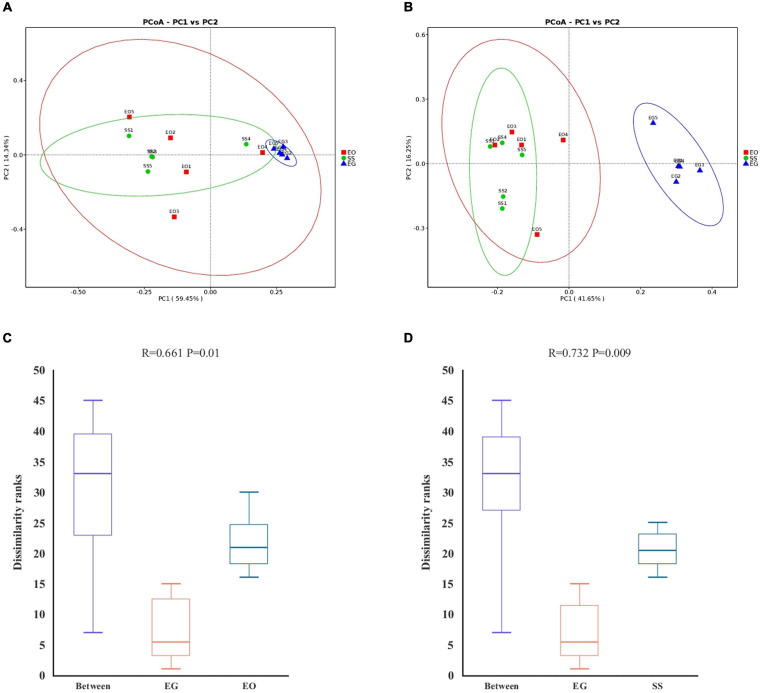
Principal component and non-parametric test analysis of bacterial diversity. Principal coordinate analysis (PCoA) of bacteria using distance based on weighted **(A)** and unweighted **(B)** UniFrac values. Non-parametric test (ANOSIM) of bacterial diversity in EG compared with EO **(C)** and SS **(D)**. EG, EO, and SS refer to *E. grisescens*, *E. obliqua*, and *S. subpunctaria*, respectively.

The PCoA analysis using the distances based on weighted and unweighted UniFrac showed that the gut bacteria communities of *E. grisescens* larvae were the most distinctive (Figures 2A,B). A non-parametric test (ANOSIM), which is based on Bray–Curtis dissimilarity, indicated that the gut bacterial beta-diversity of EG was significantly different from those of EO (*R* = 0.664; *p* < 0.05) and SS (*R* = 0.732; *p* < 0.001) (Figures 2C,D). These results showed that the gut bacterial beta-diversity of *E. grisescens* larvae was significantly distinctive compared with those of *E. obliqua* and *S. subpunctaria* larvae.

**FIGURE 3:**
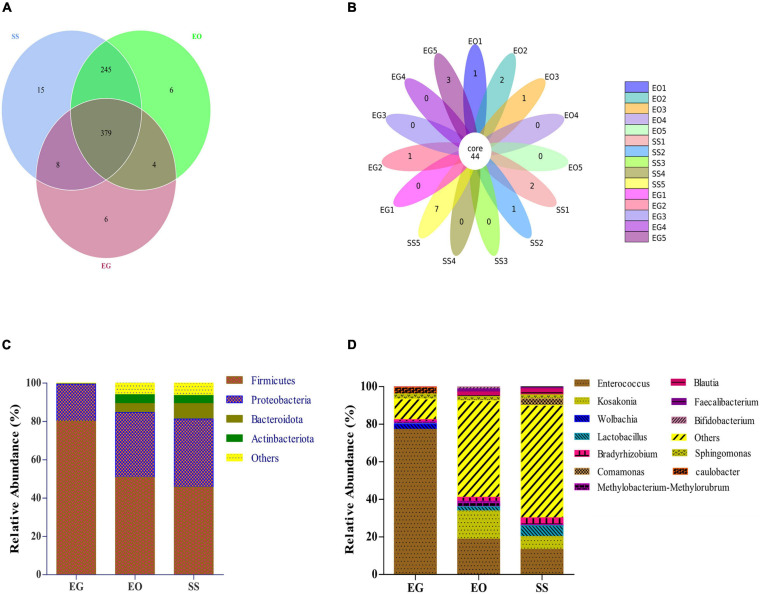
Venn diagram **(A)** and petal diagram **(B)** of operational taxonomic units (OTUs) in EG, EO, and SS. Relative abundances are shown for bacteria with relative abundance of >1% in larval gut of at least one species at the phylum level **(C)** and three species at the genus level **(D)**. EG, EO, and SS refer to *E. grisescens*, *E. obliqua*, and *S. subpunctaria*, respectively.

As shown in the Venn diagram in Figure 3A, 379 OTUs were shared by all groups, and accounted for 95.47, 59.78, and 58.58% of the total OTUs in EG, EO, and SS, respectively. The petal diagram in Figure 3B shows that a total of 44 OTUs were shared among all samples. The relative abundance of these 44 OTUs in EG, EO, and SS was 83.41% ± 6.95%, 60.01% ± 11.02%, and 65.46% ± 13.21%, respectively (Supplementary Figure 1). The alpha- and beta-diversity indexes were lowest for bacterial community in the gut of *E. grisescens* larvae, but overall, the composition of core gut bacteria in the three geometrids was similar.

The dominant bacterial phyla in each group were Firmicutes (45.64% ± 12.25% to 80.13% ± 8.63%) and Proteobacteria (19.26% ± 4.22% to 33.92% ± 4.22%) (Figure 3C). The dominant bacterial genera in EG were *Enterococcus* (77.29% ± 16.53%), *Caulobacter* (3.58% ± 1.6%), and *Wolbachia* (2.8% ± 1.52%). The dominant bacterial genera in EO and SS were *Enterococcus* (13.40% ± 5.51% to 18.8% ± 6.20%), *Kosakonia* (6.87% ± 2.33% to 14.99% ± 4.16%), and *Lactobacillus* (2.27% ± 2.33% to 5.74% ± 4.16%) (Figure 3D). These results showed that *Enterococcus* was the dominant gut bacterial genus in tea Geometridae larvae.

**FIGURE 4:**
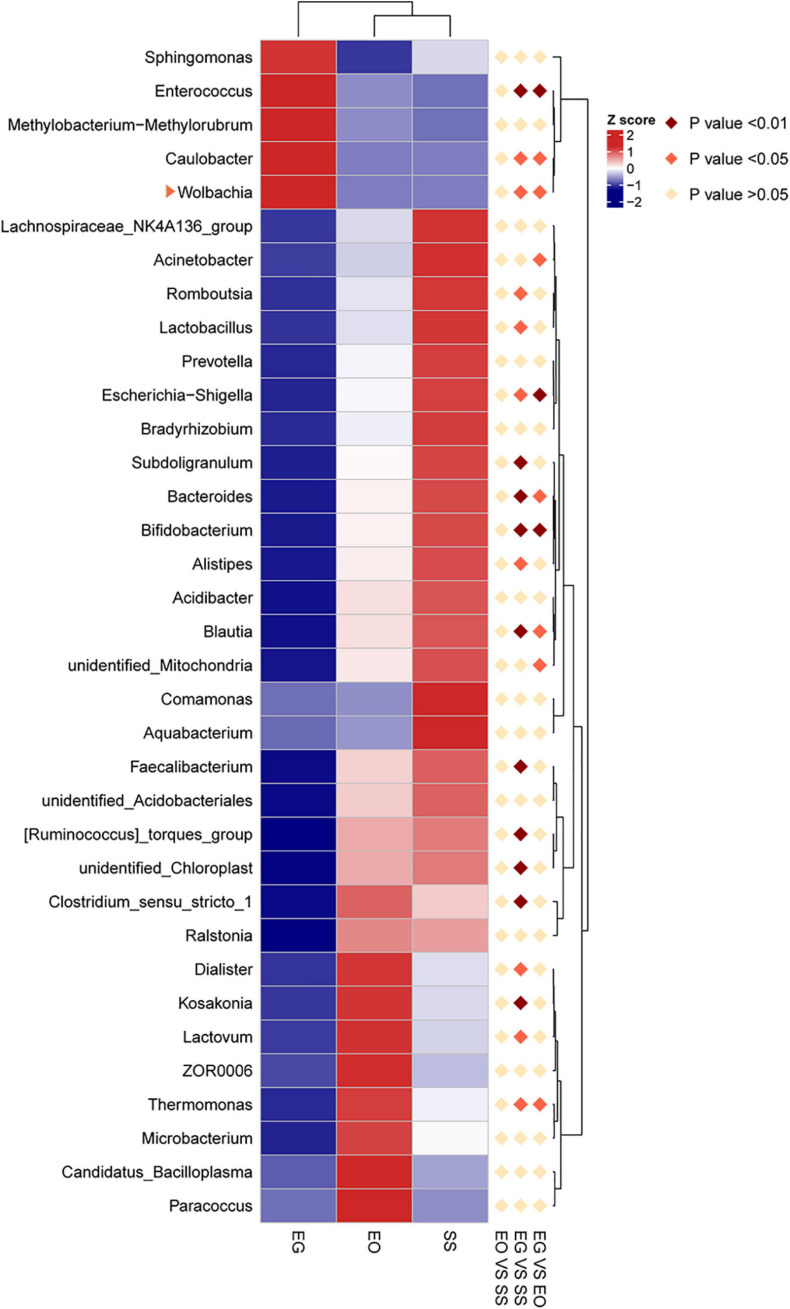
Hierarchical clustering analysis of three groups based on the relative abundance of the top 30 microbiota genera identified in EG, EO, and SS. EG, EO, and SS refer to *E. grisescens*, *E. obliqua*, and *S. subpunctaria*, respectively.

The heat map of the top 35 abundant gut bacteria illustrates differences in microbial community structures at the genus level (Figure 4). The relative abundance of *Caulobacter* and *Wolbachia* in *E. grisescens* was significantly different from that in *E. obliqua* and *S. subpunctaria* as determined by MetaStat analysis. Among these three bacterial species, *Wolbachia* was only detected in *E. grisescens* larvae, with a relative abundance of up to 7.6% (Supplementary Figure 2).

### Composition of Microbial Communities in Adult Female Moths of *E. grisescens*

The Illumina NovaSeq sequencing of bacteria from female adults of *E. grisescens* yielded about 6.33–6.73 G raw data per sample. After quality filtering and read merging, a total of 6.32–6.73 G clean data remained per sample. After removing the host’s genomic data, only 0.32–0.39 G data per sample remained for analysis. We also used a read-based (mapping) approach for metagenomic analysis.

**FIGURE 5:**
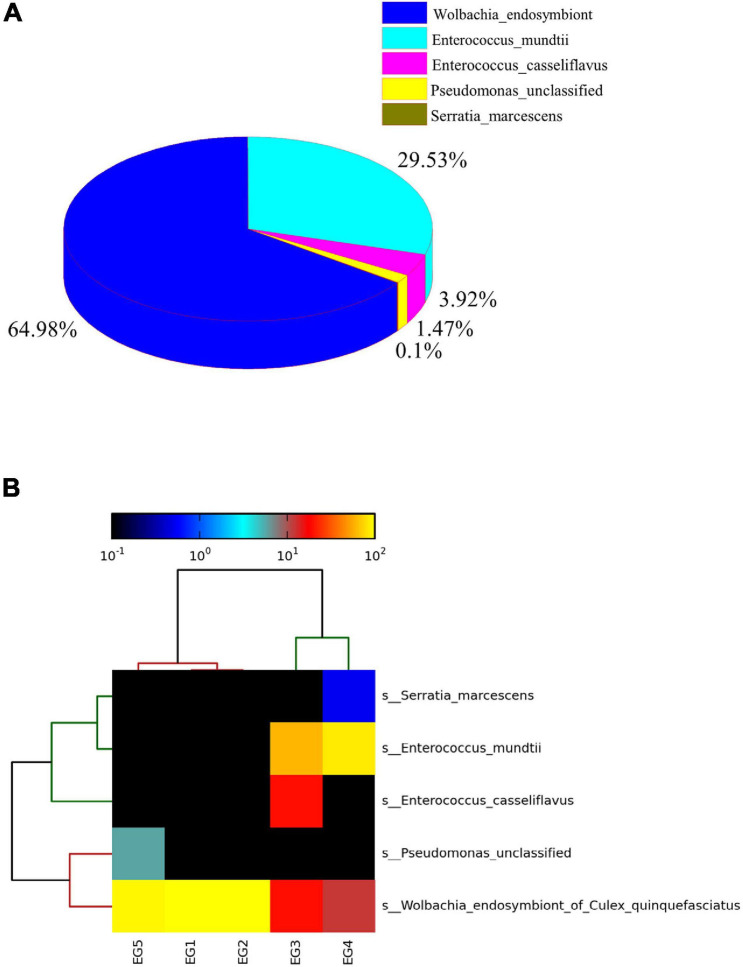
Relative abundances of microbiota species in *E. grisescens* female moths **(A)**. Hierarchical clustering analysis of five samples identified in *E. grisescens* female moths **(B)**.

The dominant bacterial species in EG female moths were *Wolbachia* endosymbiont (64.98%) and *E. mundtii* (29.35%) (Figure 5A). *Wolbachia* spp. were detected in all five EG samples and were the most abundant bacteria in EG1, EG2, and EG5 (Figure 5B).

### Molecular Characterization of Native *Wolbachia* Strain

Next, molecular typing analyses were conducted to identify the *Wolbachia* strain in *E. grisescens*. Five *Wolbachia* strains were isolated from *E. grisescens* collected from Anhui, Hubei, Zhejiang, Fujian, and Yunnan (the five representative tea-producing provinces in China) and were designated as *w*GriAH, *w*GriHB, *w*GriZJ, *w*GriFJ, and *w*GriYN, respectively.

**FIGURE 6:**
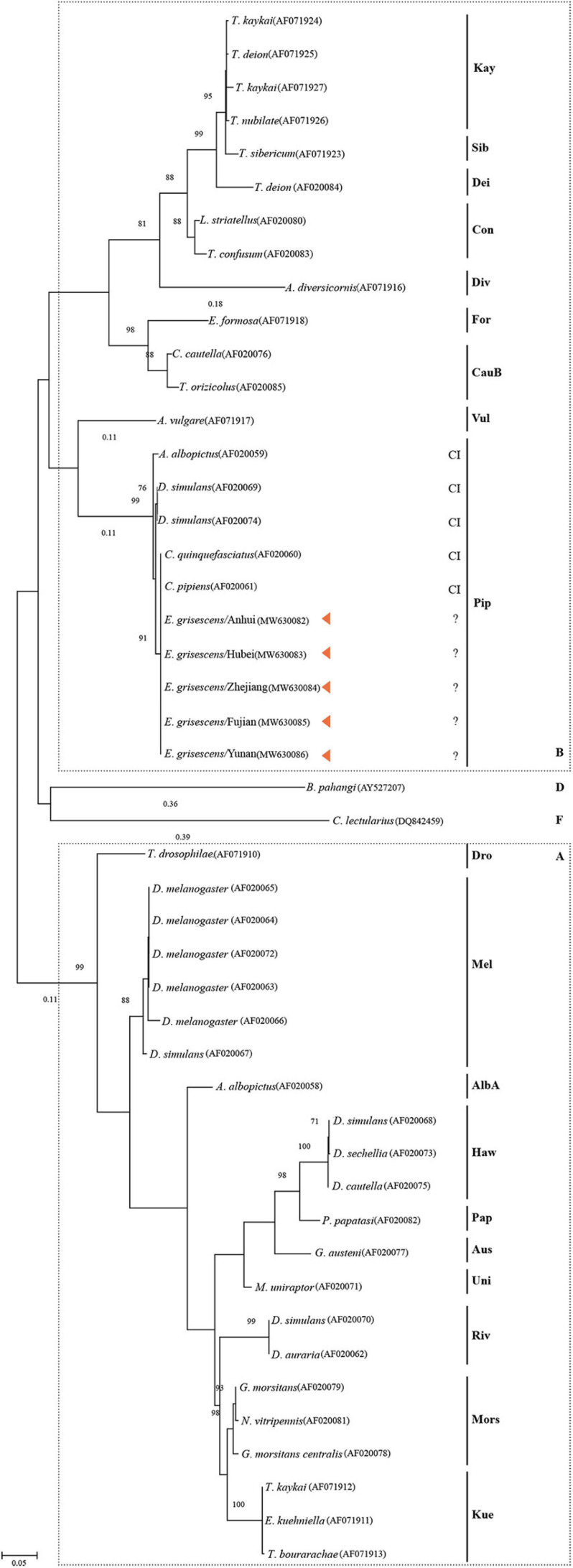
Phylogenetic tree of *Wolbachia* based on the *wsp* sequences. The tree has been constructed by the Maximum likelihood method. The name of the host arthropod species is listed in Supplementary Table 1. Bar represents amino acid substitutions per site. Bootstrap values are shown at each node. CI, cytoplasmic incompatibility; A, supergroup A; B, supergroup B; D, supergroup D; F, supergroup F.

Figure 6 shows the tree based on the general data set using the ML implementation after bootstrapping 1000 times. The topology shows the division of *Wolbachia* into four supergroups, A, B, D, and F. *w*GriAH, *w*GriHB, *w*GriZJ, *w*GriFJ, and *w*GriYN were in supergroup B, subgroup Pip. All the reference members of this group are CI-inducing strains (Figure 6).

In the MLST typing analyses, *w*GriAH, *w*GriHB, *w*GriZJ, *w*GriFJ, and *w*GriYN had the same allelic profile and HVR profiles. The allelic profile for g*atB*, *coxA*, *hcpA*, *ftsZ*, and *fbpA* was 39, 14, 40, 36, and 4 (ST-41), respectively, and the HVR profiles for HVR1, 2, 3, and 4 were 10, 8, 10, and 8 (*wsp*-10), respectively (Table 3).

**TABLE 3:** MLST and WSP HVR profiles of *Wolbachia* strains.

Strain	MLST						WSP				
	***gatB***	***coxA***	***hcpA***	***ftsZ***	***fbpA***	**ST**	**HVR1**	**HVR2**	**HVR3**	**HVR4**	***wsp***
*w*GriAH	39	14	40	36	4	41	10	8	10	8	10
*w*GriHB	39	14	40	36	4	41	10	8	10	8	10
*w*GriZJ	39	14	40	36	4	41	10	8	10	8	10
*w*GriYN	39	14	40	36	4	41	10	8	10	8	10
*w*Gri FJ	39	14	40	36	4	41	10	8	10	8	10

**FIGURE 7:**
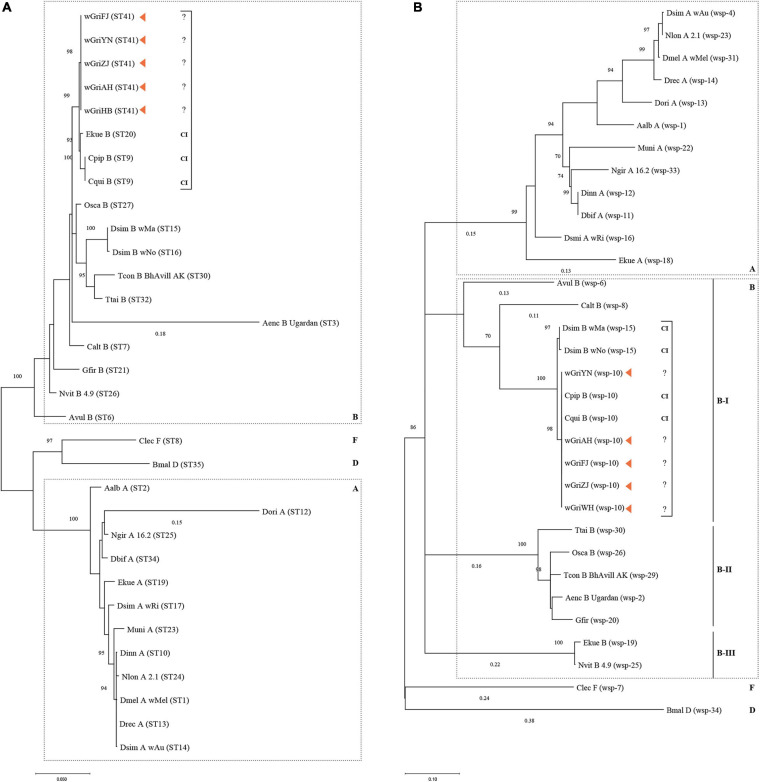
Phylogenetic analysis based on *Wolbachia* MLST and *wsp* sequences. Maximum likelihood trees constructed using the MLST allelic **(A)** and WSP HVR **(B)** profiles. For strain names, see Supplementary Table 2. Bar represents amino acid substitutions per site. Bootstrap values are shown at each node. CI, cytoplasmic incompatibility; A, supergroup A; B, supergroup B; D, supergroup D; F, supergroup F.

The MLST-based ML tree separated the isolates into three major clusters: A, B, and D + F (Figure 7A). Within supergroup B, *w*GriAH, *w*GriHB, *w*GriZJ, *w*GriFJ, and *w*GriYN, as one subclade, formed a cluster with Ekue_B, Cpip_B, and Cqui_B, all of which are CI-inducing strains. Differently, the HVR-based ML tree separated the isolates into five major clusters: A, B-I, B-II, and B-III, and a cluster containing D and F (Figure 7B). *w*GriAH, *w*GriHB, *w*GriZJ, *w*GriFJ, and *w*GriYN formed a subclade with Cpip_B and Cqui_B and placed in B-I. All the available reference *Wolbachia* strains shared the same HVR and had CI-inducing phenotypes (Figure 7B).

Based on this, *Wolbachia* isolates *w*GriAH, *w*GriHB, *w*GriZJ, *w*GriFJ, and *w*GriYN were identified as the same strain, designated as *w*Gri. Thus, *w*Gri was classified as a potentially CI-inducing *Wolbachia* strain in supergroup B.

### Effects of *Wolbachia* on *E. grisescens* Reproduction

To explore the effects of *Wolbachia* strain *w*Gri on *E. grisescens* reproduction, we treated *E. grisescens* with antibiotics to eliminate *w*Gri and carried out reciprocal cross analyses. To eliminate the effects of antibiotic interference on the cross results, *E. obliqua* and *S. subpunctaria* were treated with the same antibiotics.

**FIGURE 8:**
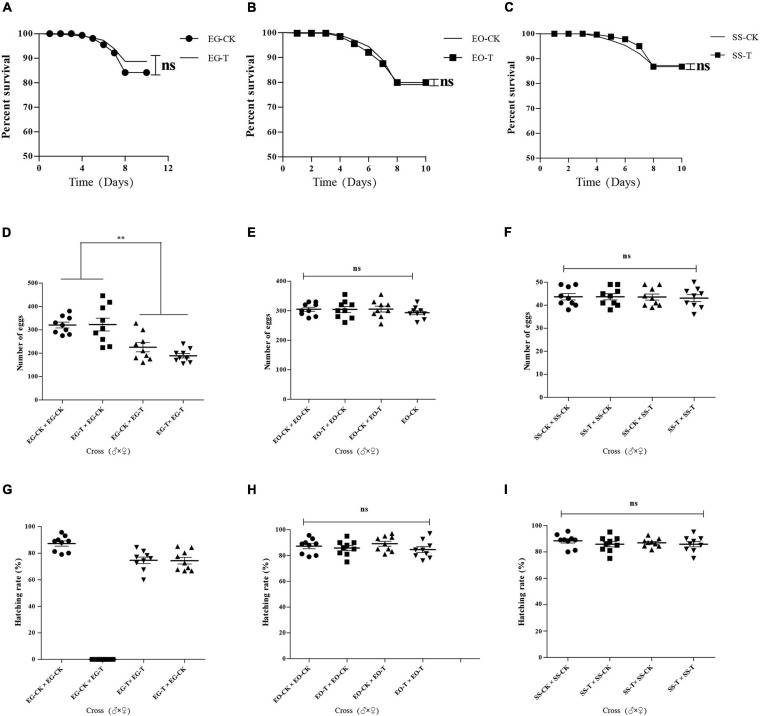
Effect of antibiotic on the survival rates of EG **(A)**, EO **(B)**, and SS **(C)**; fecundity of EG **(D)**, EO **(E)**, and SS **(F)**; and fertility of EG **(G)**, EO **(H)**, and SS **(I)**. EG, EO, and SS refer to *E. grisescens*, *E. obliqua*, and *S. subpunctaria*, respectively. Significant differences were detected by unpaired two-tailed *t*-test; ns: no significant difference; **p < 0.001.

The survival rates of *E. grisescens* infected with *Wolbachia* (EG-CK) or treated with antibiotics (EG-T) showed no significant difference (unpaired and two-tailed *t*-tests; *p* > 0.5) (Figure 8A). Egg production (fecundity) was significantly greater in infected females (♂ × ♀; EG-CK × EG-CK: 320.56 ± 35.13 eggs per female and EG-T × EG-CK: 322.56 ± 77.48 eggs per female) than in uninfected females (♂ × ♀; EG-T × EG-CK: 225.67 ± 54.99 eggs per female and EG-T × EG-T: 189.12 ± 26.64 eggs per female) in both tests (Figure 8D). This indicated that the presence of *w*Gri increased the number of eggs produced.

The fertility (total number of eggs hatched) rates of *E. grisescens* infected with *Wolbachia* (EG-CK) or treated with antibiotics (EG-T) were not significantly different (unpaired and two-tailed *t*-tests; *p* > 0.5). However, the uninfected females mated with infected males showed 0% eggs hatched per female (100% embryo mortality). Infected females mated with uninfected males had high fertility rates (Figure 8G). These results indicated that *Wolbachia* strain *w*Gri induces strong unidirectional CI in *E. grisescens*.

The survival (Figures 8B,C), fertility (Figures 8E,F), and fecundity rates (Figures 8H,I) of *E. obliqua* and *S. subpunctaria* showed no significant difference between the control and the antibiotic treatments, confirming that antibiotics did not affect the results of the crossing analyses.

These data indicated that *w*Gri resulted in a high CI level in *E. grisescens* and enhanced the fecundity of the female hosts.

### Reciprocal Cross Analysis

**FIGURE 9:**
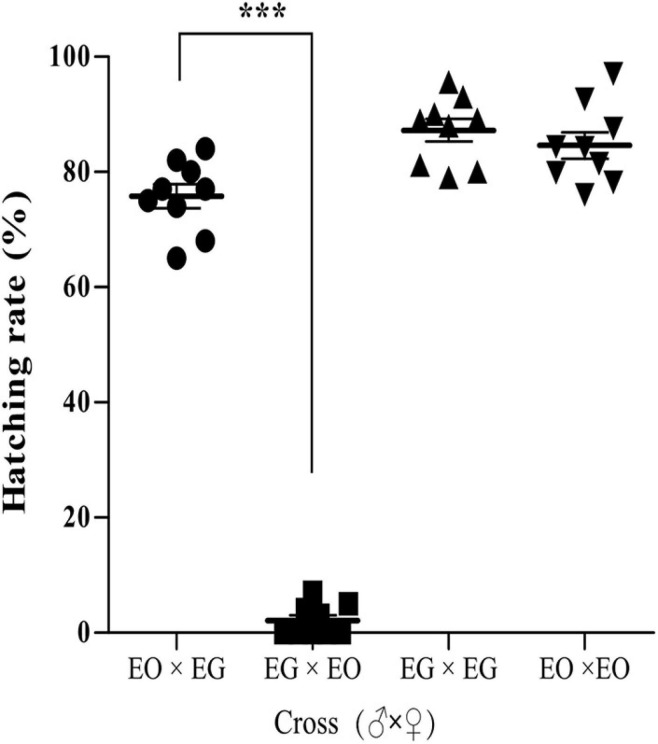
Crossing experiments between *E. grisescens* (EG) and *E. obliqua* (EO). Significant differences were detected by unpaired two-tailed *t*-test; ***p < 0.0001.

Crossing experiments were conducted using *E. grisescens* and *E. obliqua*. The hatching rate of the eggs produced by *E. grisescens* females mated with *E. obliqua* males was 75%, while the eggs produced by *E. obliqua* females mated with *E. grisescens* males did not hatch, or very few hatched (Figure 9).

These findings indicated that *Wolbachia* strain *w*Gri influences reproductive communication between *E. grisescens* and *E. obliqua* through CI.

## Discussion

The microbiomes of insects play important roles in mediating their health and fitness, and hence, microbiomes are attracting the attention of entomologists (Oliver et al., 2010; Engel and Moran, 2013; Blow and Douglas, 2019). In this study, a culture-independent approach was used to compare the composition and diversity of gut bacteria among three tea Geometridae moths, *E. grisescens*, *E. obliqua*, and *S. subpunctaria*. The results revealed that *Enterococcus* was the dominant bacterial genus in the gut of the three Geometridae larvae. Many studies have reported that *Enterococcus* is active in the gut throughout the larval life cycle of most Lepidoptera and probably plays a key role in insect defense against potentially harmful microorganisms (Dillon and Dillon, 2004; Chen B. S. et al., 2020). The results of the metagenomic analyses demonstrated that *E. mundtii* was the main *Enterococcus* species in the gut of *E. grisescens*.

Although the alpha- and beta-diversity indexes of gut bacteria were lowest in *E. grisescens* larvae, the composition of core gut bacteria (except for *Wolbachia*) was similar among the three Geometridae species. *Wolbachia* are the most widespread endosymbiotic bacteria among arthropods. In these hosts, they are notorious for their reproductive parasitism, which ensures their spread, even though it may lower host fitness (Vavre et al., 2002). For instance, CI provides the infected females with a reproductive advantage in the population, allowing *Wolbachia* to rapidly expand in the host population (Werren et al., 2008). In this study, we detected *Wolbachia* in all the tested *E. grisescens* samples collected from five different representative tea plantations. Molecular typing analyses demonstrated that these *Wolbachia* were the same strain, designed as *w*Gri. Our analyses showed that *w*Gri has a strong capability to induce CI. Besides, in a previous study, *Wolbachia* were detected in all the tested *E. grisescens* samples that had been collected from eight different geographical tea-producing areas in China (Wang et al., 2020). Consequently, we speculate that the 100% *w*Gri infection rate of *E. grisescens* in nature has resulted in the spread of *w*Gri in *E. grisescens via* induced unidirectional CI.

*Wolbachia* also have the potential to engage in mutualistic relationships with their hosts (Zug and Hammerstein, 2015). In insects, there are a few cases whereby *Wolbachia* act as an obligate mutualist. For example, in the bed bug *Cimex lectularius*, elimination of *Wolbachia* (supergroup F) through antibiotic treatment renders abnormal development of eggs, which can be restored by dietary supplementation of synthesizes biotin and riboflavin (Hosokawa et al., 2010; Nikoh et al., 2014). *Wolbachia* also act as facultative mutualists whereby hosts benefit from infection; they do not depend on *Wolbachia* for survival or fecundity (Kaur et al., 2021). The results of the mating experiment showed that *w*Gri could enhance the fecundity of its host, indicative of a mutually beneficial symbiosis between *w*Gri and *E. grisescens*. Meanwhile, elimination of *w*Gri through antibiotic treatment did not affect its host survival rate. Based on this, we hypothesize that *Wolbachia* strain *w*Gri as the facultative symbiont of its host *E. grisescens* has contributed to its rapid spread in tea gardens by enhancing its fecundity.

The tea Geometrid moths *E. grisescens* and *E. obliqua* are closely related species that feed on tea leaves. In China, *E. grisescens* and *E. obliqua* are treated as the same species in tea garden management because their morphology is so similar that it is difficult to tell them apart (Wang et al., 2019). According to the records, *E. obliqua* has always been one of the main tea pests in China. Previous data showed that it was distributed in the main tea-producing areas, while *E. grisescens* was mainly distributed in southern tea-producing areas (Luo et al., 2017). However, recent surveys have found that the distribution of *E. obliqua*, which was once distributed in all tea-producing areas, has greatly reduced. In contrast, *E. grisescens*, once a relatively minor pest species, is now distributed in almost all tea-producing provinces in China (Jiang et al., 2014; Li et al., 2019). These sibling species can mate. Our results showed that mating between *E. grisescens* and *E. obliqua* conformed to the phenomenon of unidirectional CI. In other words, the eggs produced by female *E. grisescens* mated with male *E. obliqua* are able to hatch. The characteristics of *Wolbachia* maternal inheritance have promoted rapid evolution of its host. We suggest that the gradual decrease in the distribution of *E. obliqua* and the gradual increase in the distribution of *E. grisescens* is related to the presence of *Wolbachia* strain *w*Gri.

The results of this study show that *Wolbachia* strain *w*Gri results in a high level of CI in *E. grisescens* and enhances the fecundity of its female hosts. We propose that the presence of *w*Gri in its host *E. grisescens* has contributed to host rapid spread among tea gardens by improving the host’s fertility and interfering with the reproduction of its competitors (*E. obliqua*).

## Data Availability Statement

The datasets presented in this study can be found in online repositories. The names of the repository/repositories and accession number(s) can be found below: https://www.ncbi.nlm.nih.gov/genbank/, MW630082-MW630111 and https://www.ncbi.nlm.nih.gov/, PRJNA720281.

## Author Contributions

YY, YZ, and YlZ designed the research. YL supervised the study. YZ, SL, RJ, CZ, and TG performed the experiments. YZ analyzed and wrote the manuscript. YZ, RJ, and CL revised the manuscript. All authors approved the final version for submission.

## Conflict of Interest

The authors declare that the research was conducted in the absence of any commercial or financial relationships that could be construed as a potential conflict of interest.

## Footnotes

1 http://pubmlst.org/wolbachia/

2 http://metastats.cbcb.umd.edu/

3 https://github.com/cjfields/readfq

4 http://bowtie-bio.sourceforge.net/bowtie2/index.shtml
