# Supplementary material for: Wolbachia Strain wGri From the Tea Geometrid Moth Ectropis grisescens Contributes to Its Host’s Fecundity
Source: Front Microbiol. 2021 Jul 19;12:694466. doi: 10.3389/fmicb.2021.694466 (PMC8326765; doi:10.3389/fmicb.2021.694466)
Supplement: Supplementary file 3 [file Data_Sheet_1.docx]

***Supplementary Material***

**Supplementary Table S1.**

The *wsp* gene proﬁles of reference *Wolbachia* strains.

**Supplementary Table S2.**

MLST and WSP HVR proﬁles of reference *Wolbachia* strains.

**Supplementary Figure S1**

Relative abundances of 44 OTUs in 15 samples.

**Supplementary Figure S2**

Relative abundances of *Wolbachia* in *Ectropis grisescens*
